# Lithotomy

**Published:** 1842-01

**Authors:** 


### LITHOTOMY.

Why is it that so many children of the West have urinary calculi? The four latter operations for lithotomy which we have had an opportunity of witnessing, were on small boys. The last was under four years of age, and had shown signs of calculus for 10 months. The operation was performed by Professor Gross, before the Medical Class, in the Amphitheatre of the Louisville Hospital. The calculus weighed half an ounce, and belonged to the mulberry species. It is nine days since the operation, and the little patient is doing well.

